# Effects of Nitrogen Fertilizer Types on the Uptake and Translocation of PFAS and Metabolomic Activities of Hydroponically Cultivated Lettuce (*Lactuca sativa*)

**DOI:** 10.1021/acs.jafc.5c02015

**Published:** 2025-04-28

**Authors:** Olatunbosun Adu, Syeda Sharmin Duza, Virender K. Sharma, Xingmao Ma

**Affiliations:** † Department of Water Management and Hydrological Science, Texas A&M University, College Station, Texas 77843, United States; ‡ Program for the Environment and Sustainability, Department of Environmental and Occupational Health, School of Public Health, Texas A&M University, 212 Adriance Lab Rd., 1266 TAMU, College Station, Texas 77843, United States; § Department of Chemical, Environmental, and Materials Engineering, University of Miami, Coral Gables, Florida 33146, United States; ∥ Department of Civil and Environmental Engineering, Texas A&M University, College Station, Texas 77843, United States

**Keywords:** PFAS accumulation, lettuce, nitrogen fertilizers, emerging PFAS, metabolic pathway

## Abstract

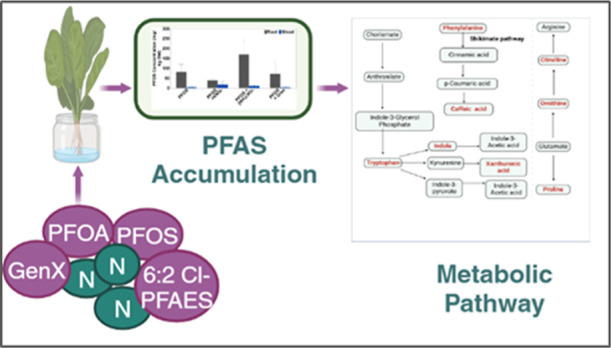

Increasing evidence is showing that per- and polyfluoroalkyl substance (PFAS) replacing compounds are not as safe as they were assumed. This study investigated the plant uptake of two most prevalent PFAS, perfluorooctanoic acid (PFOA) and perfluorooctane sulfonic (PFOS) and their replacing compounds, hexafluoropropylene oxide dimer acid (HFPO-DA or GenX) and 6:2 chlorinated polyfluoroalkyl ether sulfonates (Cl-PFESAs) (6:2 Cl-PFAES) by hydroponically grown lettuce in the presence of different nitrogen fertilizers including ammonium sulfate ((NH_4_)_2_SO_4_), potassium nitrate (KNO_3_), and urea in a growth chamber. Interestingly, (NH_4_)_2_SO_4_ significantly increased GenX uptake in lettuce shoots by ∼108% and KNO_3_ increased the uptake of PFOS and 6:2 Cl-PFAES by 267% and 395%, respectively, suggesting that the nitrogen fertilizer type has a marked impact on the plant uptake of PFAS and that this impact was PFAS-dependent. Our results also showed that (NH_4_)_2_SO_4_ generally resulted in higher PFAS root to shoot transfer than the other two types of nitrogen fertilizers. Metabolomic analysis revealed that amino acids, nucleotides, and antioxidants are the major metabolites that were either significantly increased or decreased in the lettuce shoots. These results showed that nitrogen fertilization management can have a significant impact on PFAS plant uptake and transport, and the impact depends on the types of nitrogen fertilizer and PFAS compounds.

## 1 Introduction

Per- and poly fluoroalkyl substances (PFAS) are well-known emerging environmental pollutants due to their persistence, bioaccumulation, and toxicological impacts on humans and the ecosystem.^1^ However, due to their resistance to heat, water, oil, acid, and high surface activity, PFAS applications in consumer and industrial products remain popular, even though intensive efforts are underway to develop alternative compounds to replace legacy PFAS.^2^ This has led to increasing detection of PFAS alternatives with features reasonably comparable to legacy PFAS.^3^ In agricultural soils, the main sources of PFAS could come from firefighting foams, wastewater treatment plants, landfill leachates, and land-applied biosolids.^4^

Legacy PFAS such as perfluorooctanesulfonic acid (PFOS) and perfluorooctanoic acid (PFOA) are being substituted by 6:2 chlorinated polyfluoroalkyl ether sulfonates (Cl-PFESAs) and hexafluoropropylene oxide dimer acid (HFPO-DA or GenX), respectively, because they are more hydrophilic and less bioaccumulative and were presumed to be safer. Unfortunately, recent evidence has shown that these alternatives are also highly toxic and exposure to these emerging PFAS can cause similar liver, kidney, nervous and immune system damages as those legacy PFAS. Previous studies have demonstrated the strong potential of PFAS accumulation in food crops, which may represent an important pathway of human exposure.^5−7^ Thus, it is crucial to investigate the uptake of both emerging and legacy PFAS to monitor their potential human exposure through food consumption.^8^

Nitrogen management is a routine but critical agricultural practice to improve soil fertility and augment plant yield.^9^ Overfertilization can occur when nutrients are not properly managed, causing elevated accumulation of emerging contaminants such as PFAS in edible tissues which could pose risks to human health.^10^ Currently, ammonium, nitrate, and urea are predominant nitrogen sources.^11^ These types of nitrogen may affect PFAS uptake differently by altering soil pH or other properties, for example, ammonium nutrients may acidify the soil while nitrate may increase alkalinity in the soil.^12^ Additionally, nitrogen fertilization could promote microbial activity, the plant biomass, and water uptake differently, which may impact PFAS availability for plants.^12^ However, no investigation has been performed to evaluate the effect of nitrogen fertilizer types on plant PFAS uptake.

Metabolite detection in plants provides molecular-level insights into the response of plants to diverse stress conditions.^13^ PFAS uptake in plants can impact metabolic pathways by inducing oxidative stress and trigger cellular damage such as enzyme inactivation, modification of genes, and degradation of proteins.^14,15^ For example, previous findings have shown that when lettuce (*Lactuca sativa*) was exposed to 500 and 5000 ng/L of PFOA and PFOS for 28 days, there was a significant upregulation of the lipids, carbohydrates, fatty acids, amino acids, and antioxidants. Additionally, there was an interference in the major pathways responsible for energy metabolism.^15^

Lettuce was used in this study because it is a popular food crop and has been commonly used in previous studies on PFAS plant uptake.^16,17^ However, most previous studies focused on only legacy PFAS and no studies have explicitly investigated the impact of nutrient management on plant PFAS uptake. The objectives of this study included (i) determining the plant uptake and accumulation of the two most prevalent legacy PFAS (PFOA and PFOS) and their replacing compounds (GenX, 6:2 Cl-PFAES), (ii) investigating the impact of nitrogen fertilizer types on the plant uptake and accumulation of concerned PFAS compounds, and (iii) gaining molecular-level insight into changes of metabolite profiles of lettuce shoots after exposure to concerned PFAS in the presence of different nitrogen fertilizers, including ammonium sulfate, potassium nitrate, and urea. In most field operations, ammonium in the pore water of top soil can range from a few micrograms per liter to hundreds of milligrams per liter, depending on the crop species, amount and frequency of fertilizer application, and soil properties.^18^ A hydroponic system was chosen for this study to avoid the compounding effects of soil such as the microbial activity in soil.^8^ In addition, the increase in urban agriculture has led to an increasing adoption of hydroponic systems.^8^

## 2 Materials and Methods

### 2.1 Chemicals and Materials

PFOA (99%), PFOS (99%), ammonium sulfate (>99%), potassium nitrate (>99%), urea (99–100.5%), and ammonium acetate were obtained from Sigma-Aldrich (St. Louis, MO, USA) and GenX and 6:2 Cl-PFAES were obtained from Wellington Laboratories (Guelph, ON, Canada). Modified Hoagland solution mixture was purchased from USBiologic (MA). Acetone and methanol were purchased from Thermo Fisher (MA). Tetrabutylammoniumhydrogensulfate (TBAHS) and *tert*-butyl methyl ether (MTBE) were obtained from Acros Organics (Geel, Belgium). Sodium carbonate, sodium hydroxide, and ammonium hydroxide solution were purchased from Thermo-Fisher (MA). All reagents used were HPLC (high performance liquid chromatography) grade. Oasis WAX cartridges (30 mg, 1 cm^3^) were purchased from Waters Co. (Milford, MA, USA). LC/MS (liquid chromatography–mass spectrometry)-grade methanol and chloroform were purchased from Thermo Fisher (MA, USA).

### 2.2 Hydroponic Experiment

Lettuce seeds were purchased from Johnny’s Selected Seeds (Winslow, ME, USA). They were sterilized for 10 min using a 2% bleach solution (Chlorox, CA) and then rinsed thoroughly with deionized (DI) water before germination. The seeds were left to germinate on a wet filter paper in disposable Petri dishes for 4 days, under a 16 h/8 h day-to-night cycle at room temperature (25–30 °C). After germination, the seedlings were transferred to 50 mL falcon tubes filled with 1/4 strength modified Hoagland solution and wrapped in foil to prevent light exposure. Plants were grown for 28 days, and the tubes were topped with hydroponic solution when needed. Afterward, the roots of the seedlings were thoroughly rinsed with ultrapure water and transferred to new 50 mL falcon tubes with different treatment solutions. An image of the experimental setup is provided in Figure S1. There were 17 treatments with 10 replicates for each treatment for this study including: 500 μg/L of PFOA, PFOS, GenX, 6:2 Cl-PFAES, and a combination of the 500 μg/L of the different PFAS with 300 mg/L of three different types of nitrogen fertilizers as well as a control (containing pure water only). The detailed treatment scenarios are summarized in Table S1.

Assuming that 1 kg of soil contains approximately 180 g water as in most field growing conditions,^19^ the N concentration in the pore water was calculated at ∼277.8 to 694.4 mg N/L, at the typical nitrogen fertilization rates between 50 and 125 mg N/kg soil.^20^ Therefore, 300 mg of N/L was chosen for our study, which falls within the typical range of N concentration in the pore water when nitrogen fertilizers are first applied. The treatment solutions were replenished every 48 h, and the volume added to each tube for replenishment was documented. After 7 days of treatment exposure, the plants were removed from the solution and the roots were thoroughly rinsed with ultrapure water. The plants were then separated into roots and shoots to determine their fresh weight. Three replicates were stored in a freezer at −20 °C for 24 h prior to the use of the freeze-drying process to determine the PFAS concentration in the plant tissues. The remaining seven replicates of the shoot tissues were stored at −80 °C for metabolomic analysis.

### 2.3 PFAS Extraction in Plant Tissues

The extraction for PFAS followed a previously published method^21^ with minor modification. Briefly, the freeze-dried biomass lettuce shoots and roots were grounded and 4.0 mL of 0.4 M NaOH was mixed with the biomass. The mixture was stored in a refrigerator at 4 °C overnight. A 2.0 mL portion of tetrabutylammonium hydrogensulfate (TBAHS, 0.5 M) and 4.0 mL of Na_2_CO_3_ buffer (0.25 M) were then added to the mixture. The mixture was vortexed and then 5 mL of *tert*-butyl methyl ether (MTBE) was added, and the mixture was shaken for 20 min. The organic and aqueous layers were separated by centrifugation at 4500 rpm for 5.0 min and the MTBE layer (top layer) was transferred to a second polypropylene tube. The left-over mixture was extracted twice with 5.0 mL MTBE each time and MTBE fractions of all three extractions (∼15 mL) were collected. ^13^C-PFHxA (^13^C-labeled perfluorohexanoic acid) was used as an extracted internal standard to determine the extraction efficiency.

The samples were allowed to evaporate with nitrogen gas purging and then reconstituted into 0.10 mL of methanol and diluted with 0.9 mL of deionized water, followed by solid phase extraction by passing the sample through a preconditioned Water Oasis Wax 1 cm^3^ Vac cartridge (Thermo Scientific, Waltham, MA, USA) with 1.0 mL of 0.1% NH_4_OH in methanol and 1.0 mL of 100% methanol, and this was repeated thrice. A 1.0 mL of the sample was added immediately after conditioning the cartridges. Then, 1.0 mL of 25 mM ammonium acetate was added to wash the cartridge which was allowed to dry under low vacuum for 5.0 min. Once the cartridge had dried fully, 1.0 mL of 0.1% NH_4_OH in methanol (eluent) was added under very low vacuum pressure, the eluent was transferred from the glass collection tubes to HPLC vials with inserts, and the concentration of PFAS in the sample was determined by a binary pump high-performance liquid chromatography coupled to a triple quadrupole mass spectrometer (Thermo Fisher Scientific, Waltham, USA) (LC–MS/MS) following EPA method 1633.

### 2.4 Metabolomic Analysis in Lettuce Shoots

Analysis on water-soluble metabolites in plant tissues was conducted using liquid chromatography high resolution accurate mass (LC-HRAM). Freshly harvested lettuce shoot tissues were stored in 50 mL polystyrene Falcon tubes and lyophilized overnight at −80 °C. A 50.0 mg amount of lettuce shoot tissue was preweighed in a Precellys 24 tissue homogenization tube and extracted twice with the addition of 800 μL ice cold methanol/chloroform (1:1, v/v) containing 0.25 μg/mL internal standard (l-leucine), followed by homogenization for 30 s on intensity of 6000 rpm and then centrifugation at 15,000 rpm for 10 min at 4 °C. The supernatants were transferred to a clean 15.0 mL polystyrene centrifuge tube and stored on ice. 600 μL of HPLC grade water (ice cold) was added to the extract, which was then vortexed for 30 s and centrifuged at 4000 rpm for 10 min at 4 °C. Afterward, 500 μL of 0.2 μm filtrate was pipetted to a 3 kDa MWCO Amicon Ultra filter placed in a collection tube, and the samples were centrifuged at 15,000*g* for 1 h at 4 °C. Analysis of the samples was performed using LC-HRAM.

### 2.5 Statistical Analysis

Experimental data were reported as the mean (±standard deviation). Statistical analyses were performed by one-way ANOVA using the JMP software. Tukey’s test was performed for posthoc comparison, and differences were considered significant when *p* ≤ 0.05, with letters denoting significant groups. To investigate the similarities between metabolites, a correlation heat map was generated using the R package.

## 3 Results and Discussion

### 3.1 Biomass of Lettuce

**Figure 1:**
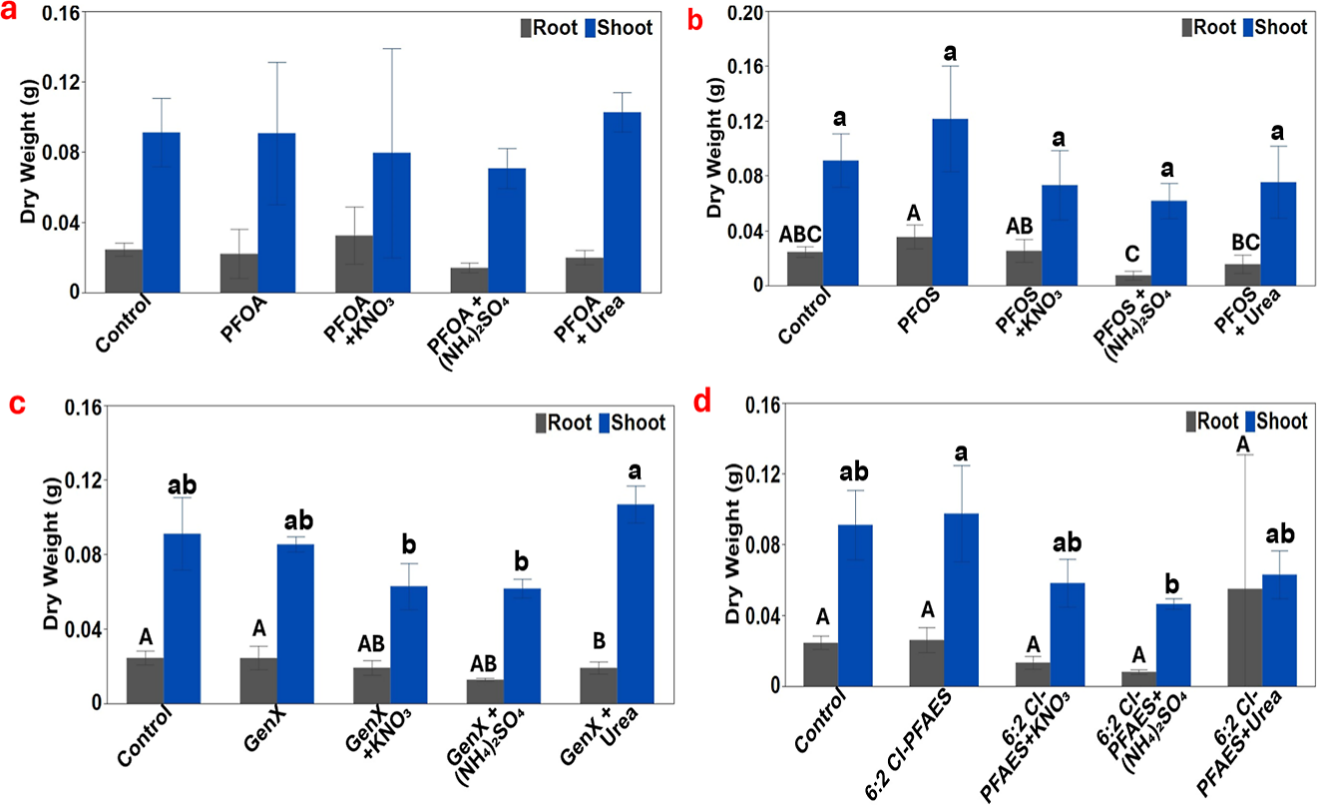
Root and shoot dry weight of lettuce tissues under different treatments of 0.5 mg/L of PFAS. (a) PFOA, (b) PFOS, (c) GenX, and (d) 6:2 Cl-PFAES and different combinations of KNO_3_ or (NH_4_)_2_SO_4_ and urea. Values represent mean ± SD (*n* = 3). Different letters indicate significant differences (*p* ≤ 0.05) according to one-way ANOVA, followed by Tukey’s test. Letters were not shown when significant differences were not observed.

As shown in Figures 1 and S2, the lettuce root and shoot biomass exposed to PFOA both in the presence and absence of different nitrogen fertilizers was not significantly different from the control plants even though the shoot biomass from the treatment PFOA + (NH_4_)_2_SO_4_ and PFOA + KNO_3_ was insignificantly reduced by 22.4% and 12.7% and the root biomass from the treatment of PFOA + (NH_4_)_2_SO_4_ and PFOA + urea was insignificantly reduced by 42.3% and 18.8% compared to the control. The PFOS alone or in the presence of different nitrogen fertilizers also did not significantly impact the shoot biomass. However, PFOS in the presence of either (NH_4_)_2_SO_4_ or urea significantly reduced the root biomass by 69.7% and 36.6%, respectively, compared to the PFOS alone treatment. Different from the legacy PFAS, the lettuce shoot biomass was more notably affected by the two replacing PFAS compounds in the presence of nitrogen fertilizers. For example, GenX + KNO_3_ and GenX + (NH_4_)_2_SO_4_ reduced the shoot biomass by 31.0% and 32.3%, respectively, compared to the control, and the shoot biomass from these treatments was significantly lower than the GenX + urea treatment. Similarly, 6:2 Cl-PFAES in the presence of (NH_4_)_2_SO_4_ markedly reduced the shoot biomass by 49.0% compared with the control. While it is surprising that none of the nitrogen fertilizers significantly enhanced the lettuce shoot biomass in this short-term study, our results highlighted the importance of nitrogen management, necessitating a comprehensive long-term study in the future. The lack of enhancive effect of nitrogen fertilizers in this study was likely due to the sufficient nutrient the seedlings contained from their prior growth media (Hoagland solution) before they were exposed to PFAS and additional nitrogen fertilizers.

### 3.2 Accumulative Water Transpiration of Lettuce

**Figure 2:**
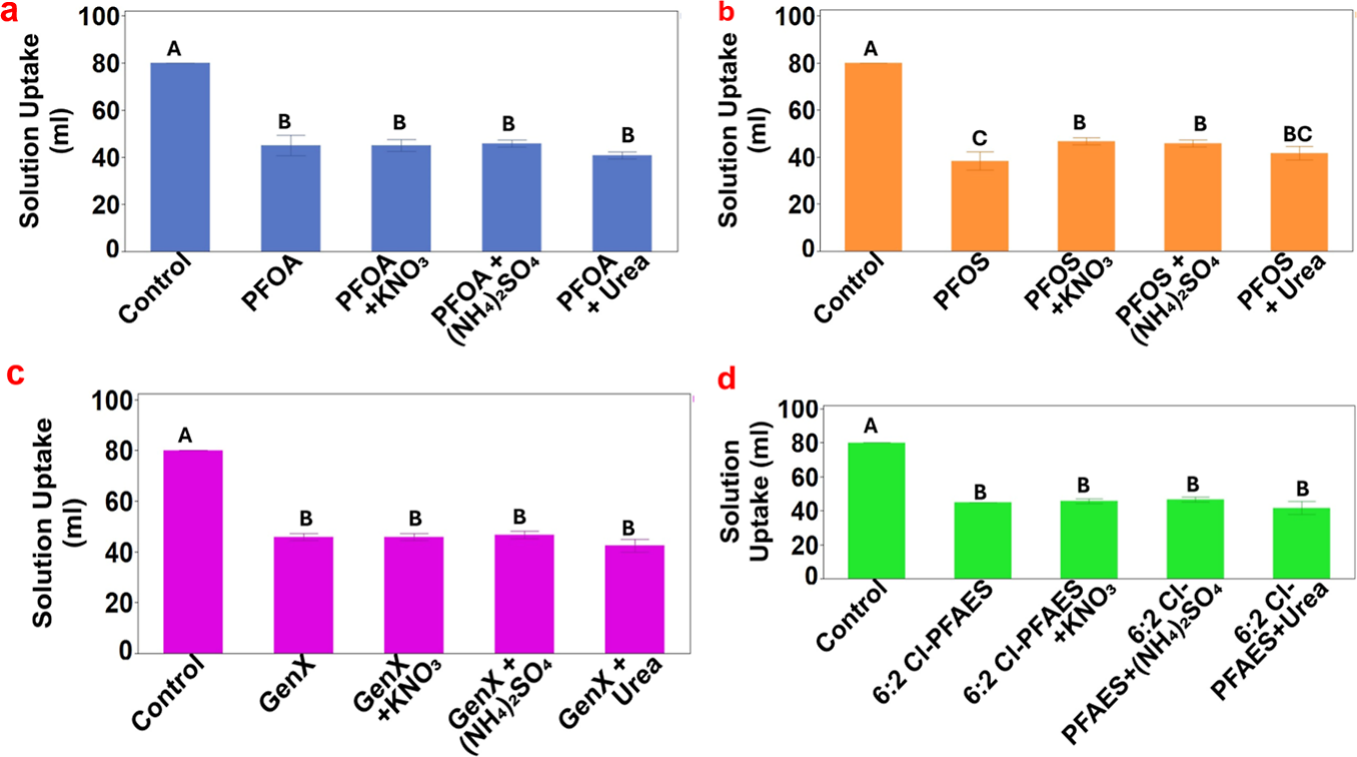
Accumulative water transpiration of lettuce seedlings under different treatments of 0.5 mg/L (a) PFOA, (b) PFOS, (c) GenX, and (d) 6:2 Cl-PFAES in the presence of KNO_3_, (NH_4_)_2_SO_4_, or urea. Values represent mean ± SD (*n* = 3). Different letters indicate significant differences (*p* ≤ 0.05) according to one-way ANOVA, followed by Tukey’s test.

Consistent with the generally lower biomass in the presence of PFAS and nitrogen fertilizers, the accumulative water transpiration from different treatments showed that the exposure to PFAS at the level of this study significantly lowered plant water uptake regardless of the presence or absence of nitrogen fertilizers, as shown in Figure 2. For most PFAS compounds, the supply of nitrogen fertilizer did not improve the plant water uptake. However, the addition of KNO_3_ or (NH_4_)_2_SO_4_ significantly increased the accumulative water transpiration by 21.7% and 19.6% compared with the plant exposed to PFOS alone, which significantly decreased the accumulative water transpiration by 52.1% compared with the control.

### 3.3 Uptake and Accumulation of PFAS by Lettuce

**Figure 3:**
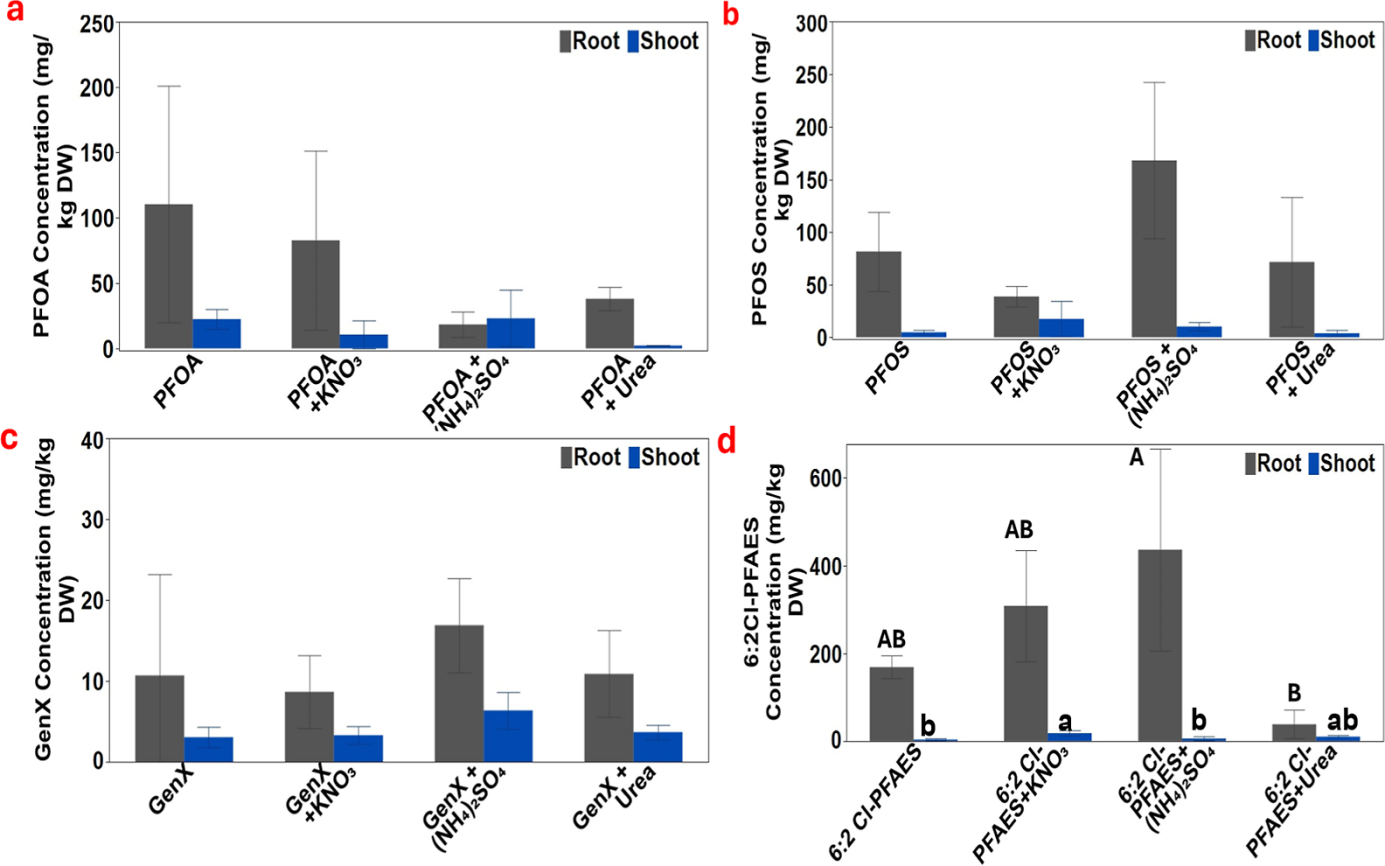
PFAS Accumulation in lettuce root and shoot tissues under different treatments of 0.5 mg/L of PFAS. (a) PFOA, (b) PFOS, (c) GenX, and (d) 6:2 Cl-PFAES and different combinations of KNO_3_ or (NH_4_)_2_SO_4_ and urea. Values represent mean ± SD (*n* = 3). Different letters indicate significant differences (*p* ≤ 0.05) according to one-way ANOVA, followed by Tukey’s test. Letters were not shown when no significant differences were observed.

PFAS accumulation in edible tissues of plants represents a significant pathway for human exposure. Therefore, the uptake and accumulation of PFAS in lettuce tissues in the presence or absence of different nitrogen fertilizers were closely examined. As shown in Figure 3, even though the presence of nitrogen fertilizers generally did not exhibit statistically significant differences in PFAS accumulation in lettuce shoots compared with the PFAS alone treatment based on one-way ANOVA analysis, the differences in PFAS concentrations in lettuce tissues were nonetheless noticeable. For example, the presence of KNO_3_ and urea markedly decreased PFOA uptake by 52.5% and 65.6% compared to PFOA alone treatment, but (NH_4_)_2_SO_4_ showed a minimal impact on PFOA accumulation in lettuce shoots. The supply of growth media with KNO_3_ and (NH_4_)_2_SO_4_ significantly increased the PFOS accumulation in lettuce shoots by 266.6% and 114.7% compared to PFOS alone, while urea insignificantly decreased the PFOS concentration in lettuce shoots by 12.2%. Compared with PFOA, GenX generally had lower concentrations in lettuce shoots regardless of the treatments. Unlike PFOA, the provision of (NH_4_)_2_SO_4_ with GenX significantly increased GenX uptake in lettuce shoots by 108.5% compared to GenX alone. KNO_3_ had a minimal impact on the GenX concentration in lettuce shoots. Surprisingly, urea notably increased the GenX concentration in lettuce shoot by 20.5% compared with the GenX treatment alone. Compared with PFOS, 6:2 Cl-PFAES demonstrated higher potential to accumulate in lettuce shoots, consistent with our previous study.^8^ Different from other PFAS compounds, the presence of all nitrogen fertilizers appreciably increased the shoot concentration of 6:2 Cl-PFAES, with KNO_3_ significantly increased its concentration by 395.5% while (NH_4_)_2_SO_4_ and urea insignificantly increased its shoot concentration by 56.9% and 172.3% compared to 6:2 Cl-PFAES alone treatment.

The presence of nitrogen fertilizers also markedly affected the PFAS root concentration in lettuce. All three nitrogen fertilizers noticeably decreased PFOA root concentration compared to the PFOA treatment alone. In combination with the elevated PFOA concentration in the presence of (NH_4_)_2_SO_4,_ our results suggested that ammonia-N may substantially enhance the root-shoot transfer of PFOA. In contrast to PFOA, the presence of (NH_4_)_2_SO_4_ greatly increased the root concentration of PFOS by 649.7% but did not result in higher root to shoot transfer. The result agrees with previous studies that PFOA and PFOS use different transporters for their plant root uptake and in planta transport.^22^ The nitrogen fertilizers in general showed relatively minor effects on GenX plant root uptake and the root to shoot transfer of GenX, but both KNO_3_ and (NH_4_)_2_SO_4_ notably increased the concentration of 6:2 Cl-PFAES in lettuce root, while urea substantially lowered its concentration.

Clearly, our results demonstrated that nitrogen fertilization management can markedly affect PFAS plant uptake and transport, and the specific impact would vary with the types of nitrogen fertilizer and PFAS compounds. Nitrogen fertilization is critical for healthy plant growth in agriculture because it affects a range of physiological and biochemical processes in plants as well as the expression of protein transporters.^23^ Our results provide a new perspective in minimizing PFAS uptake by properly selecting nitrogen fertilizers while providing adequate nutrients for plant growth.

### 3.4 Metabolic Response of Lettuce Shoots to Different Treatments

**Figure 4:**
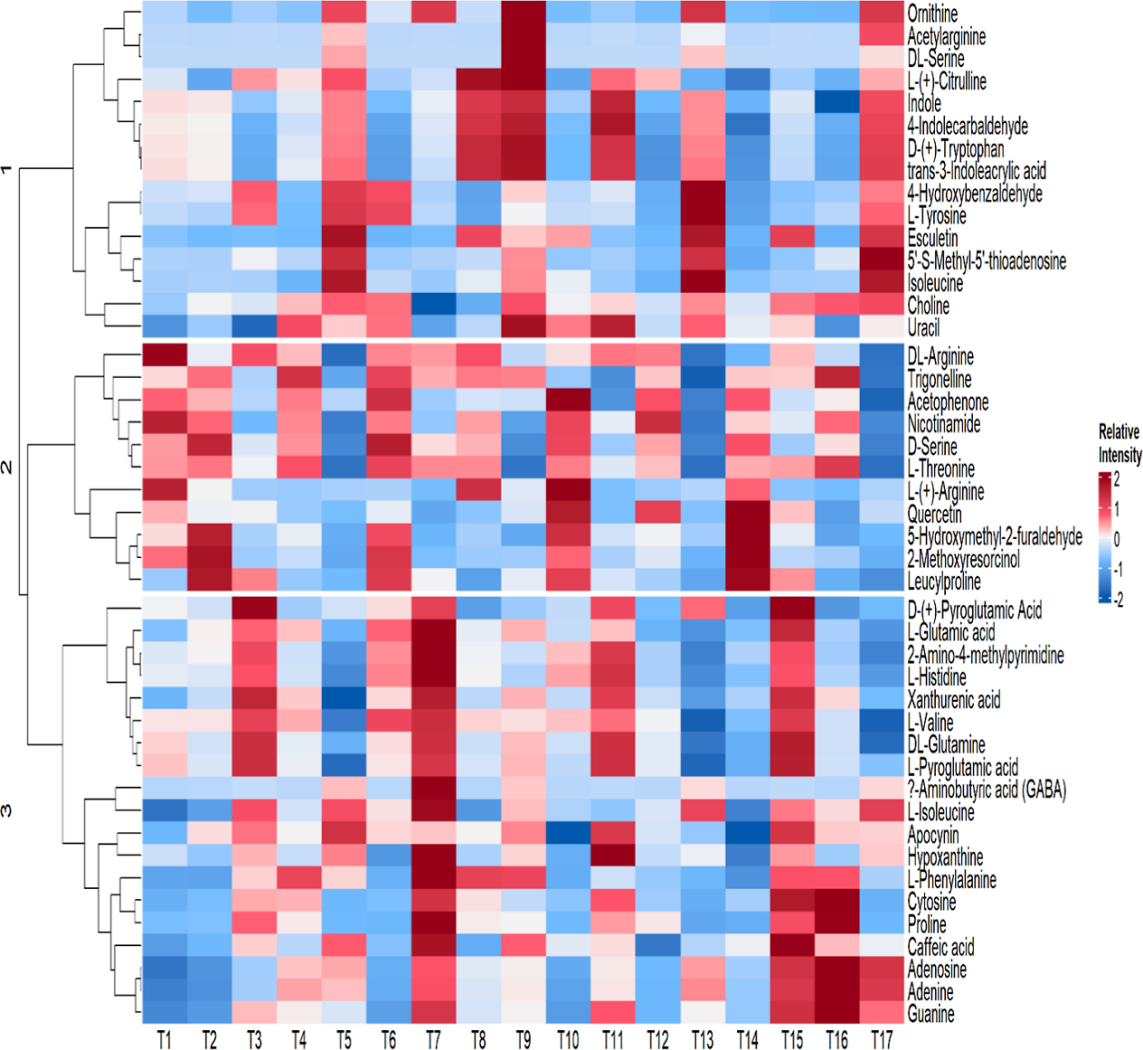
Heat map produced by hierarchical analysis of metabolites showing different treatments. The color scale signifies the normalized peak area from LC-HRAM listed for the metabolites. T1 to T17 represent the different treatments: T1: control; T2: 6:2 Cl-PFAES; T3: 6:2 Cl-PFAES + (NH_4_)_2_SO_4_, T4: 6:2 Cl-PFAES + KNO_3_, T5: 6:2 Cl-PFAES + urea, T6: GenX, T7: GenX + (NH_4_)_2_SO_4_, T8: GenX + KNO_3_, T9: GenX + urea, T10: PFOA, T11: PFOA + (NH_4_)_2_SO_4_, T12: PFOA + KNO_3_, T13: PFOA + urea, T14: PFOS, T15: PFOS + (NH_4_)_2_SO_4_, T16: PFOS + KNO_3_, and T17: PFOS + urea.

To gain more molecular insights into the different impacts of nitrogen fertilizer types on plant PFAS uptake and transport, metabolomic analysis on lettuce shoot was conducted. A total of 15,023 compounds were identified, and about 45 of these were detected as plant-relevant metabolites that showed significant differences between treatments. The metabolites identified include amino acids, antioxidants, nucleotides, and dipeptides, which are linked to nitrogen metabolism, stress response, and signaling in plants. In Figure 4, the hierarchical analysis showed three different clusters of metabolites among treatment groups. The first cluster of metabolites was upregulated in GenX + urea followed by PFOA + urea and PFOS + urea compared to the control. Nonproteinogenic amino acids such as ornithine significantly increased by 276.2% to 546.3% in the presence of urea, compared to the control, while there was a significant decrease, ranging from 26.9% to 71.4% in treatments of PFAS alone or PFAS in the presence of two other inorganic nitrogen fertilizers. For l(+)-citrulline, there was a significant increase in all 6:2 Cl-PFAES or GenX + nitrogen fertilizer treatments except for the GenX + (NH_4_)_2_SO_4_ treatment. Legacy PFAS compounds showed different impact on the levels for l(+)-citrulline, with most treatments resulting in lower concentrations of this chemical except for the PFOA + (NH_4_)_2_SO_4_, PFOA + KNO_3_, and PFOS + urea treatment in comparison with the control. Other metabolites such as indole and tryptophan were increased significantly in various PFAS treatments compared with the control.

The second cluster of metabolites includes amino acids, dipeptides, and some metabolites involved in the oxidative stress response. Dipeptides such as leucylproline showed a decreasing trend in 6:2 Cl-PFAES + KNO_3_, 6:2 Cl-PFAES + urea, GenX, GenX + urea, PFOS ,and PFOS + urea but an increasing trend in 6:2 Cl-PFAES, 6:2 Cl-PFAES + (NH_4_)_2_SO_4_, GenX + (NH_4_)_2_SO_4_, GenX + KNO_3_, PFOS + (NH_4_)_2_SO_4_, and all PFOA treatments compared to the control. Additionally, arginine, which effectively stores nitrogen in plants because of its high nitrogen-to-carbon ratio, decreased considerably in almost all PFAS treatments except for the PFOA alone treatment and the PFOS + KNO_3_ and PFOS + urea treatments. The third cluster metabolites include nucleotides such as adenosine and adenine as well as some amino acids and antioxidants. Some treatments such as the GenX + (NH_4_)_2_SO_4_ treatment resulted in greater abundance of these metabolites compared to control, but others such as PFOS alone treatment caused lower abundance in lettuce shoots. There was also a significant increase in metabolites such as caffeic acid, xanthurenic acid, and proline in the GenX + (NH_4_)_2_SO_4_ treatment compared to the control, even though their significance for PFAS plant uptake is not clear.

**Figure 5:**
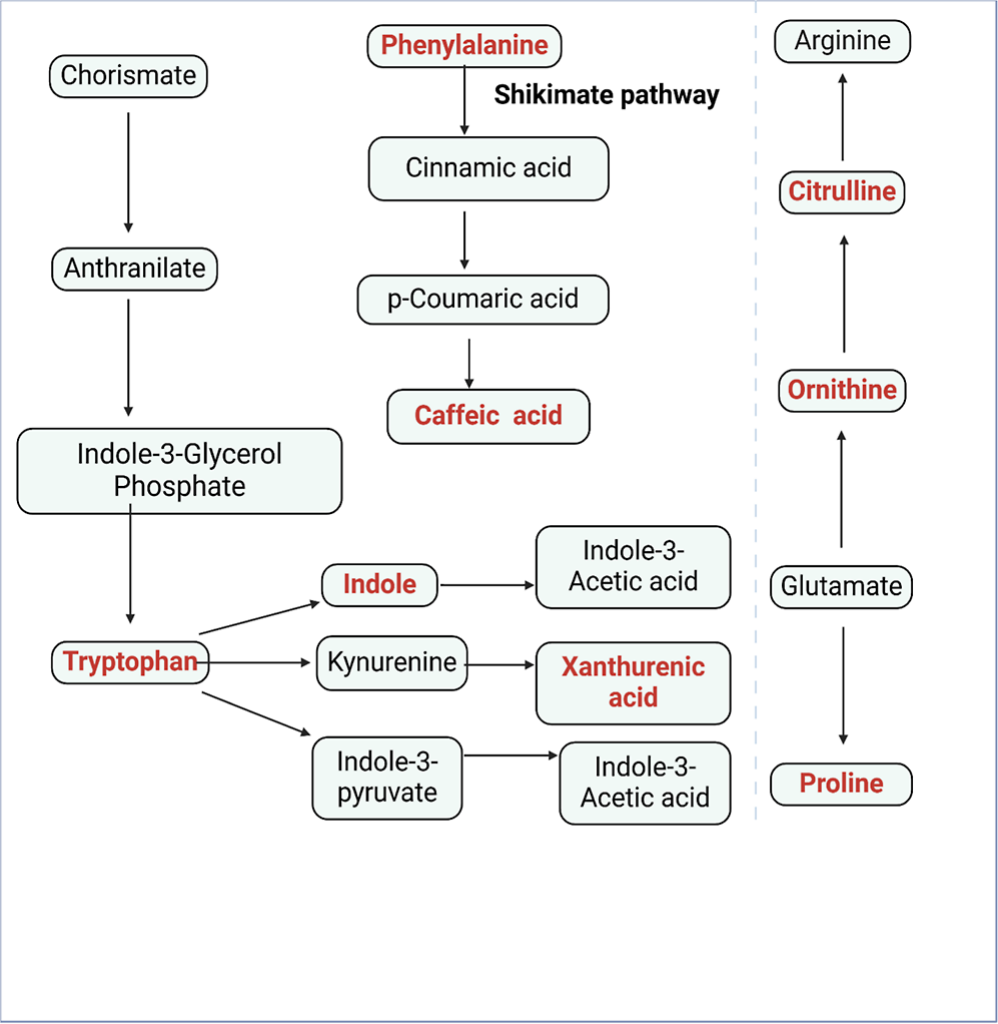
Metabolic pathways of lettuce shoot exposed to PFAS coexposure treatments. Red colors indicate the metabolites were upregulated for PFAS coexposure treatments.

The metabolic pathway of lettuce shoots to the different treatments is summarized in Figure 5. Amino acids are important to protein synthesis, signaling, and stress responses in plants.^19^ Arginine is involved in the synthesis of polyamines and plant stress responses,^19^ and significant downregulation of arginine was noticed for some treatments as explained above. The increase in proline metabolism signifies the combined metabolic activities to promote stress tolerance via nitrogen absorption, amino acid, and protein production.^19^ Ornithine and citrulline are important amino acids for the urea cycle^20^ and their combined increase with glutamate shows that there was an increase in nitrogen fixation in coping with induced stress in lettuce shoot. Phenylalanine metabolism directly controls the biosynthesis of (poly)phenols through the shikimate/phenylpropanoid pathway^21^ and phenylalanine ammonia-lyase enzyme controls this process and catalyzes the deamination of phenylalanine, producing cinnamic acid and further modifications generates hydroxycinnamic acids such as caffeic acid.^22^ The xanthurenic acid pathway in plants is primarily linked to the tryptophan metabolism, an essential amino acid.^23^ This pathway plays a crucial role in secondary metabolism, producing bioactive compounds that may have roles in plant defense and signaling.^23^

The indole pathway in plants is an important metabolic pathway that leads to the synthesis of various bioactive compounds, including tryptophan, indole-3-acetic acid (IAA), indole alkaloids, and glucosinolates.^24^ These compounds play significant roles in plant growth, development, and defense mechanisms.^24^ High nitrogen fertilization can increase the activity of some transporters [e.g., amino acid transporters, ATP-binding cassette (ABC transporters), and organic acid transporters] related to PFAS uptake.^24^ For example, previous studies have shown that ABC transporters aid in the translocation of xenobiotics or amino acids in plants which can be applied to PFAS uptake in plants.^24^ A recent study also showed that PHT1;8 protein was involved in PFOS plant uptake,^25^ further substantiating our hypothesis that proper nutrient management strategies could help restrain plant PFAS uptake.

In conclusion, our study showed that the introduction of nitrogen fertilizer may considerably impact the uptake and accumulation of PFAS in lettuce shoots, and in general, the inorganic nitrogen fertilizer tends to have greater impact than the organic fertilizer (urea) in terms of plant PFAS uptake. The specific impact on PFAS accumulation depends on the properties of both PFAS and the nitrogen fertilizers. The metabolic response of lettuce (*L. sativa*) shoots exposed to PFAS in the presence of various nitrogen fertilizers revealed that amino acids, nucleotides, antioxidants, and peptides were the major metabolites of stress response. These results offer a deeper insight into the plant response to PFAS when different nitrogen fertilizers are applied. Overall, our results demonstrated that nutrient management practices play a significant role in minimizing the risks of PFAS plant uptake and human exposure. Future studies should take into consideration the long-term exposure of plants to PFAS in the copresence of various nitrogen fertilizers so that proper guidelines may be developed to mitigate PFAS exposure via nutrient management in agricultural practices.

## Supporting Information Available

An image of the hydroponic experimental setup, root and shoot fresh weight of lettuce tissues under different treatments, chemical structure of PFAS compounds used in this study, and summary of different treatments (PDF)
