# Supplementary material for: Effects of Nitrogen Fertilizer Types on the Uptake and Translocation of PFAS and Metabolomic Activities of Hydroponically Cultivated Lettuce (Lactuca sativa)
Source: J Agric Food Chem. 2025 Apr 28;73(18):10907–13. doi: 10.1021/acs.jafc.5c02015 (PMC12063172; doi:10.1021/acs.jafc.5c02015)
Supplement: Supplementary file 1 — jf5c02015_si_001.pdf [file jf5c02015_si_001.pdf]

Supporting information

Effects of Nitrogen Fertilizer Types on the Uptake and Translocation of PFAS and Metabolomic Activities of Hydroponically Cultivated Lettuce (*Lactuca Sativa*)

Olatunbosun Adu[1], Syeda Sharmin Duza[2], Virender K. Sharma[3, *] and Xingmao Ma[4, *]

[1]Department of Water Management and Hydrological Science, Texas A&M University, College Station, TX 77843, USA
[2]Program for the Environment and Sustainability, Department of Environmental and Occupational Health, School of Public Health, Texas A&M University, 212 Adriance Lab Rd., 1266 TAMU, College Station, TX 77843, USA
[3]Department of Chemical, Environmental, and Materials Engineering, University of Miami, Coral Gables, FL, 33146, USA
Email: vks38@miami.edu
[4]Department of Civil and Environmental Engineering, Texas A&M University, College Station, TX, 77843, USA
Email: xma@civil.tamu.edu

Corresponding authors:

Xingmao Ma: xma@civil.tamu.edu

Virender K. Sharma: vks38@miami.edu

Supplementary Figure: 2

Supplementary Table: 2

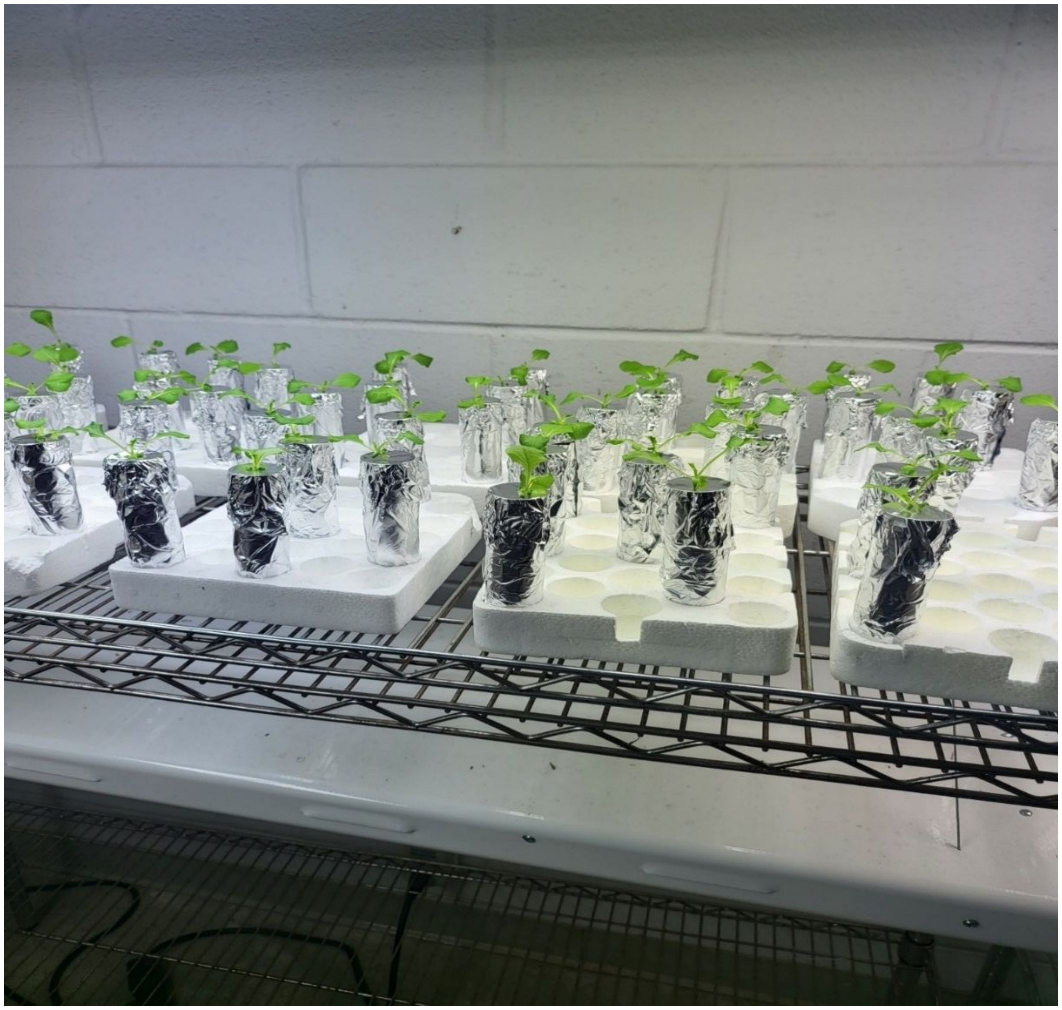

Figure S1: Picture of hydroponic setup of lettuce plants under different treatments used in this study

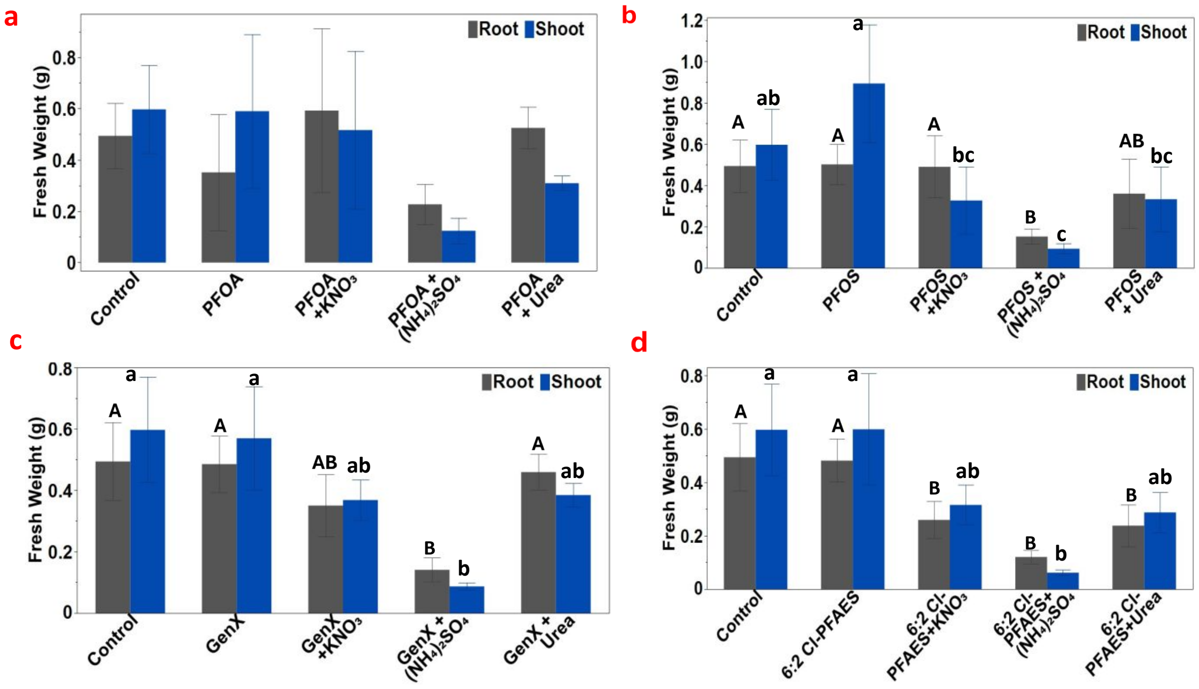

Figure S2: Root and shoot fresh weight of lettuce under different treatments of 0.5 mg/L of a) PFOA b) PFOS c) GenX d) 6:2 Cl-PFAES in the presence of 300 mg/L of KNO_3, $(NH_4)_2SO_4$ or urea. Values represent mean ± SD ($n = 3$). Different letters indicate significant differences ($p \leq 0.05$) according to one-way ANOVA followed by Tukey's test.

Table S1: Chemical Structure of PFAS compounds used in this study

PFAS Compounds	Chemical Structure
PFOA	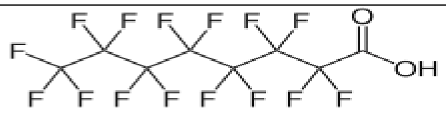
PFOS	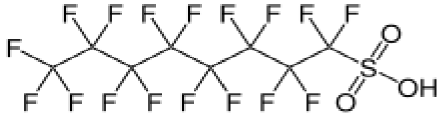
GenX	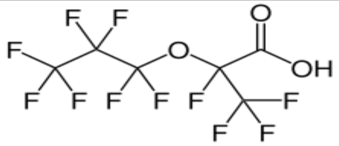
6:2Cl-PFAES	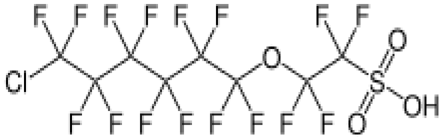

Table S2: Summary of different treatments

**Treatment**	**Description**
T1	control
T2	6:2 Cl-PFAES
T3	6:2 Cl-PFAES+ $(NH_4)_2SO_4$
T4	6:2 Cl-PFAES+KNO_3
T5	6:2 Cl-PFAES+urea
T6	GenX
T7	GenX+$(NH_4)_2SO_4$
T8	GenX+KNO_3
T9	GenX+urea
T10	PFOA
T11	PFOA+$(NH_4)_2SO_4$
T12	PFOA+KNO_3
T13	PFOA+urea
T14	PFOS
T15	PFOS+$(NH_4)_2SO_4$
T16	PFOS+KNO_3
T17	PFOS+urea
